# Neural Regulation of Cardiovascular Response to Exercise: Role of Central Command and Peripheral Afferents

**DOI:** 10.1155/2014/478965

**Published:** 2014-04-09

**Authors:** Antonio C. L. Nobrega, Donal O'Leary, Bruno Moreira Silva, Elisabetta Marongiu, Massimo F. Piepoli, Antonio Crisafulli

**Affiliations:** ^1^Department of Physiology and Pharmacology, Fluminense Federal University, Niterói, RJ, Brazil; ^2^Department of Physiology, Wayne State University School of Medicine, Detroit, MI, USA; ^3^Section of Exercise Physiology, Department of Physiology, Federal University of São Paulo, SP, Brazil; ^4^Sports Physiology laboratory Lab., Department of Medical Sciences, University of Cagliari, Italy; ^5^Heart Failure Unit, Cardiac Department, Guglielmo da Saliceto Polichirurgico Hospital, Piacenza, Italy

## Abstract

During dynamic exercise, mechanisms controlling the cardiovascular apparatus operate to provide adequate oxygen to fulfill metabolic demand of exercising muscles and to guarantee metabolic end-products washout. Moreover, arterial blood pressure is regulated to maintain adequate perfusion of the vital organs without excessive pressure variations. The autonomic nervous system adjustments are characterized by a parasympathetic withdrawal and a sympathetic activation. In this review, we briefly summarize neural reflexes operating during dynamic exercise. The main focus of the present review will be on the central command, the arterial baroreflex and chemoreflex, and the exercise pressure reflex. The regulation and integration of these reflexes operating during dynamic exercise and their possible role in the pathophysiology of some cardiovascular diseases are also discussed.

## 1. Introduction: General View of the Cardiovascular Regulation During Exercise

One of the most studied issues in integrative physiology is how circulation is regulated during exercise, a condition where mechanisms controlling the cardiovascular apparatus have to deal with two main tasks: (1) to provide adequate oxygen to fulfill metabolic demand of exercising muscles and to guarantee metabolic end-products washout; and (2) to regulate arterial blood pressure in order to maintain adequate perfusion of the vital organs without excessive pressure variations [1].

The close relation between the energetic demand of exercising muscles and cardiovascular functions is testified by the fact that during dynamic exercise cardiac output (CO) normally rises linearly as a function of O_2_ uptake [2–11]. Moreover, physical activities that involve large muscle mass, such as running and cycling, induce remarkable metabolic vasodilatation in the muscle vasculature, thereby reducing systemic vascular resistance (SVR). This fact constitutes a great challenge for the cardiovascular apparatus because it would cause a drop in blood pressure, thereby impairing brain and muscle perfusion, if control mechanisms did not contemporary augment CO. Dynamic exercise is characterized by a small increase in mean arterial pressure (MAP), whereas during static/isometric exercise there is a well-established progressive increase in MAP. This small MAP increment induced by dynamic exercise takes place despite the profound SVR reduction due to the metabolic-induced vasodilatation [3, 10, 12, 13]. The skeletal muscle releases some putative vasodilators during contractions, including potassium, adenosine, hydrogen ions, carbon dioxide, and phosphate. Moreover, an increase in pulse pressure takes place [14, 15]. This fact increases shear stress, which plays an important role in the regulation of endothelial function during exercise, such as the production of nitric oxide [16, 17], which exerts a vasodilating effect.

The increase in CO is achieved thanks to an increase in heart rate (HR) and stroke volume (SV), which counteract the reduction in SVR via a flow-increment mechanism, that is, by a rise in CO. This cardiovascular regulation is highly effective and it is remarkable that the target blood pressure can often be achieved despite a lack in response of one of these regulated variables [18–20].

It is well established that these cardiovascular responses are governed by mechanical mechanisms (skeletal-muscle and respiratory pumps), which propel blood towards the heart, thereby enhancing myocardial preload and SV, and by nervous mechanisms, which regulate vagal tone and flow in order to meet the metabolic demand of exercising muscles.

Concerning mechanical mechanisms, both respiratory and skeletal muscle pumps contribute to the increase in SV and CO occurring during dynamic exercise. It appears that, compared with the respiratory pump, the skeletal muscle pump plays the most important role since the muscle rhythmic contractions occurring during dynamic exercise create intramuscular oscillations which facilitate blood flow to the heart and enhance cardiac preload, thus increasing SV by recruiting the Frank-Starling mechanism [13, 21–26].

As regards the neural component of this regulation, exercise induces parasympathetic withdrawal and sympathetic activation, which are a function of exercise intensity and the muscle mass recruited [27, 28]. Parasympathetic withdrawal aims at increasing HR, while the engagement of the sympathetic activity aims at increasing HR, at enhancing myocardial contractility to increase SV, at inducing venoconstriction to improve venous return, at increasing vascular resistance in the abdominal viscera and nonactive skeletal muscles, and at preserving most of the available CO for the perfusion of the active muscle, where metabolic-mediated vasodilation takes place.

In this autonomic modulation some neural mechanisms are required for normal physiological response: one is a central mechanism, while the others are peripheral. In the central mechanism, commonly known as “central command”, the activation of regions of the brain responsible for motor unit recruitment also activates the cardiovascular control areas located in the medulla. It is thought that central command establishes a basal level of sympathetic activity to the cardiovascular apparatus closely linked to the intensity of the effort [29–32]. This basic pattern of autonomic activity is in turn modulated by peripheral signals originating from mechano- and metaboreceptors within the muscle, which reflexively modulate sympathetic tone on the basis of the mechanical and metabolic conditions of the contracting muscle. This latter mechanism is commonly called “exercise pressor reflex” [30, 33–36]. The sympathetic activation resulting from central command and exercise pressor reflex activation causes an enhancement in HR, in myocardial contractility, and in venous return, which together concur in raising CO. Sympathetic stimulation is in turn modulated by baroreflexes, which are reset during exercise (see Section 3 for more details). Baroreflexes oppose any mismatch between vascular resistance and CO by controlling muscle vasodilatation and cardiac chronotropism in order to avoid any excessive variation in blood pressure [33, 37, 38]. Moreover, a significant contribution of the sensory inputs from arterial chemoreceptors to sympathetic modulation during exercise has recently been demonstrated [39].

**Figure 1:**
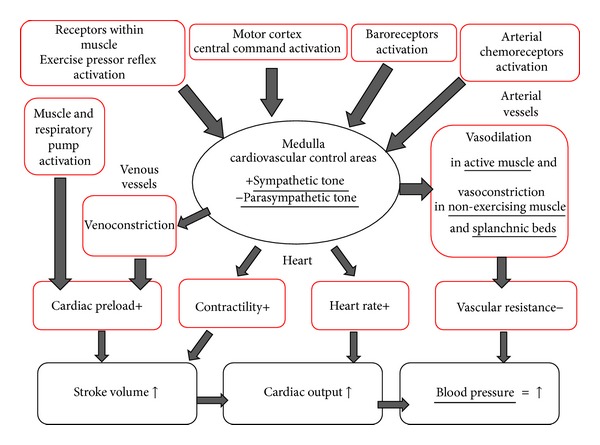
Cardiovascular adjustments during dynamic exercise. Impulses from motor cortex, contracting muscle, baroreceptors, and chemoreceptors converge on the cardiovascular control areas situated in the medulla. These controlling centers operate an integration and modulate the cardiovascular status on the basis of motor strategy, metabolic and mechanic conditions of exercising muscle, blood pressure values, and arterial gas concentration.

In short, hemodynamic status is modulated by the nervous system during exercise by integrating signals originating from the brain (central command) and from the periphery (exercise pressor reflex, baroreflexes, and arterial chemoreflex). In healthy individuals, the final result is that sympathetic activity to cardiovascular apparatus augments and prevails over parasympathetic tone, which conversely declines. As a consequence, HR and myocardial contractility increase, while there is arterial constriction in the vascular beds of organs and tissues not involved in exercise. Furthermore, there is an increase in cardiac preload, due to muscle pump activation and sympathetic-induced venoconstriction, which together contribute to the SV increment commonly observed during dynamic exercise (Figure 1).

On stopping exercise this situation is suddenly reversed: inputs from central command are reduced or even abolished (depending on whether the recovery is active or passive) and an abrupt withdrawal of the stimuli arising from muscle metabo- and mechanoreceptors occurs. This leads to a reduction in sympathetic nerve activity, while parasympathetic tone increases. As a result, HR, myocardial contractility, SV, CO, and MAP rapidly decline [24–26, 40, 41]. Moreover, at the end of effort the muscle pump reduces or even ceases its activity and cardiac filling rapidly decreases [24, 42, 43]. This fact leads to SV reduction, which would result in a decrease of MAP if compensatory events (i.e., baroreflex-mediated peripheral vasoconstriction and increased HR) did not contemporary adjust HR and SVR in order to counteract changes in SV.

In this review, we will briefly summarize neural mechanisms operating during dynamic exercise, that is, central command, baroreflex, arterial chemoreflex, and exercise pressure reflex. The main focus of the present review will be on the regulation and integration of these reflexes operating during dynamic exercise and their possible role in the pathophysiology of some cardiovascular diseases. It should be acknowledged that these reflexes work together and are not mutually exclusive in the regulation of cardiovascular response to exercise. Redundancy between them exists and neural occlusion can be operative; that is, their effects do not sum.

## 2. Central Command

The central command consists of activation of regions of the brain responsible for somatomotor activation that also impinge on the cardiovascular control areas located in the medulla, mediating autonomic responses that are crucial for cardiovascular regulation during exercise. This mechanism was first proposed by Zuntz and Geppert in 1886 for the control of ventilation [44] and by Johansson in 1893 for the control of circulation [45]. The latter conducted experiments in rabbits in Stockholm. At that time, he observed that pulse rate increased during voluntary movement, but not during passive movement or electrical stimulation. Therefore, he suggested that central motor impulses directed to the skeletal muscles irradiated to control areas governing the cardiovascular system, which was obligatory for cardiovascular responses.

Although Johansson was not correct about the contribution of afferent feedback from skeletal muscles based on subsequent studies (discussed ahead in Section 5), his hypothesis about cortical irradiation motivated later experiments conducted in humans by Krogh and Lindhard in 1913 at the University of Copenhagen, in collaboration with Miss Buchanan who worked in Oxford [46]. She likely gave a significant contribution, but she was not included as a coauthor of the study probably due to gender bias. The former collected respiratory variables, while the latter used an ECG to record HR. Both groups acquired physiological responses during leg cycling with a time resolution that is impressive for the technology available at that time. Collectively, the authors found that cardiorespiratory responses occurred slightly before, abruptly at, or with a very short latent period (i.e., 1 s) after the beginning of exercise. Therefore, they reasoned that it was not possible that any humoral factor (e.g., increase in CO_2_ produced by skeletal muscles) or temperature variation mediated the cardiorespiratory responses at the onset of exercise, and thus probably cortical irradiation to cardiorespiratory control areas was the primary mechanism responsible. Subsequently, several studies conducted in animals [47, 48] and humans [49–51], using varied experimental approaches, confirmed that their data and interpretation were correct.

In 1959, Ochwadt et al. were pioneers in the use of tubocurarine to investigate the role of central neural mechanisms in humans [52]. This drug blocks the neuromuscular junction, requiring a larger amount of central motor activation to generate a certain absolute amount of tension. They found that the initial increase in ventilation at the onset of exercise was higher during curarization than without the drug.

However, there was no control of the degree of curarization; neither authors investigated the effect on steady-state exercise. Later, in 1964, Asmussen et al. used standardized intravenous infusions of tubocurarine during steady-state leg cycling in two subjects at the University of Copenhagen [53]. After infusions, handgrip force decreased (25% to 50% in one subject; 12% to 25% in another) but ventilatory and circulatory variables did not change at rest. During exercise, infusions increased perceived effort to pedal. Pulse rate and blood pressure increased slightly and irregularly but the ventilation increased, both absolutely and as a percentage of the oxygen uptake, by up to 50%, under experimentally-induced isocapnia. The absence of change in ventilation at rest and previous evidence that the tubocurarine infusion protocol reduced neck lift (approximately 95%) and handgrip force (approximately 65%) [54], but did not change importantly the force of respiratory muscles, suggest that the curarization did not interfere importantly on the respiratory muscles. A limitation was that curarization increased the energy expenditure to perform the same amount of work, which may have enhanced the activation of muscle metaboreceptors and confounded the interpretation about the role of central command. Anyway, these results suggested that central neural mechanisms contributed to the cardiorespiratory responses to exercise in humans.

In 1972, Goodwin et al. conducted a notable study in humans at the University of Oxford [55]. They applied vibration at the distal biceps tendon during static contractions with the biceps or the triceps. The vibration intended to activate muscle spindle afferents, which provided reflex medullary excitation when applied to the agonist muscle (i.e., biceps), reducing the central motor activation to achieve a given tension. On the other hand, vibration provided reflex medullary inhibition when applied to the antagonist muscle (i.e., triceps), rising the central motor activation to achieve a given tension. The authors observed that blood pressure, HR, and pulmonary ventilation increased less when central motor activation reduced, whereas the opposite was observed when central motor activation rose. This reinforced the existence of a central neural mechanism, which authors named “central command” and that became the most common name used to describe the phenomenon in the literature.

Eldridge et al. in 1985 advanced the findings from studies in humans, via experiments in cats [56]. Preparations included anesthetized cats with intact brains, unanesthetized with decortication at the level of the hypothalamus, and unanesthetized with decerebration at level of the mesencephalon. Spontaneous actual locomotion and attempts to move (fictive locomotion; motor electrical activity in peripheral nerves after pharmacological-induced paralysis) occurred in all preparations, except the mesencephalic cats. In addition, electrical stimulation or injection of a GABA antagonist (picrotoxin) into a subthalamic region of the hypothalamus caused locomotion. In all cases when locomotion occurred, respiration and arterial pressure increased in proportion to the level of locomotor activity despite control or absence of respiratory and muscle feedback mechanisms and lack of change of metabolic rate. Hence, these data strongly supported the central command concept and suggested that neither the motor cortex nor neural or humoral feedback mechanisms are required for the central command operation.

**Figure 2:**
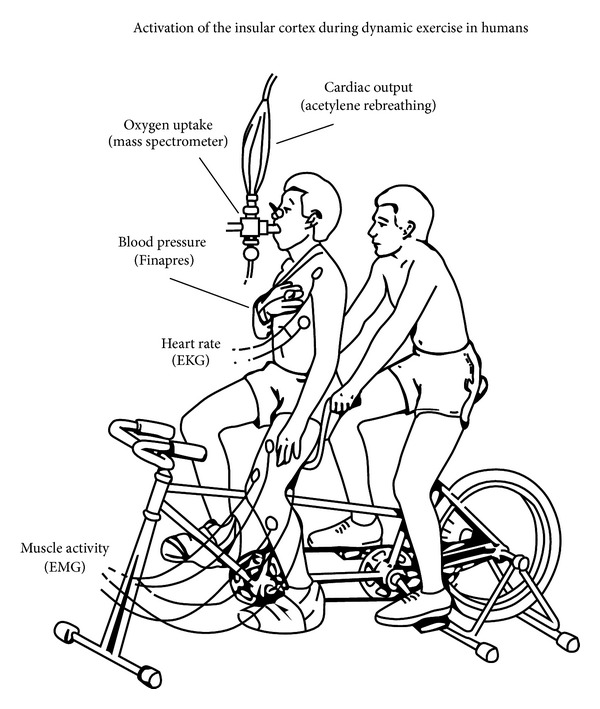
Experimental setup showing an adapted tandem bicycle. This model was used to separate the contribution of central command and mechanoreflex. Active cycling (AC) was used to activate both the central command and the mechanoreflex. Passive cycling (PC) was used to activate only the mechanoreflex. In this situation, the rider on the rear seat pedaled while the subject on the front seat kept his legs relaxed. Passive cycling was also combined with electrical stimulation (PC + ES) in some subjects, to match the oxygen consumption observed during active cycling, reproduced with permission from Williamson et al. 1997 [57].

**Figure 3:**
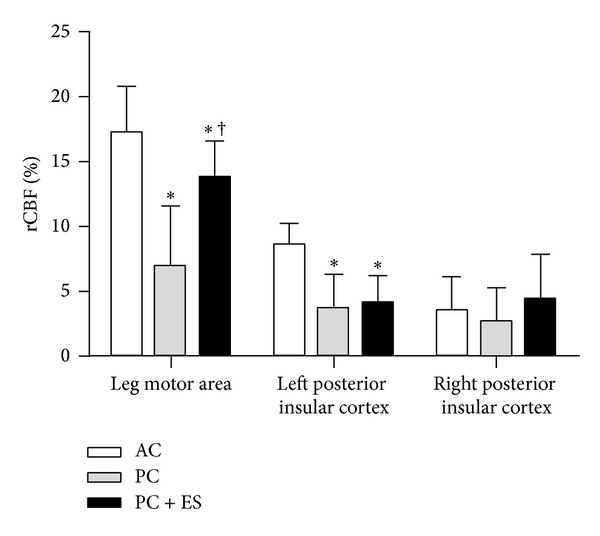
Percent increases in regional cerebral blood flow (rCBF) distribution from rest to active cycling (AC), passive cycling (PC), and passive cycling with electrical stimulation (PC + ES). Data presented as mean ± SD. ∗, *P* < 0.05 versus AC; †, *P* < 0.05 versus PC. This was the first demonstration where left insular cortex activation is involved in the central command of cardiovascular responses in humans. Moreover, findings during PC + ES supported the role of leg muscle afferent signals towards the full activation of leg motor areas, adapted from Williamson et al. 1997 [57].

Based on Eldridge et al.'s study, many other studies in animal models, and studies in humans that used neuroimaging [57, 58], deep brain electrical recording [59], or deep brain stimulation [60], it has been generally accepted that a feed forward process is involved in the central command, which can function without feedback information from skeletal muscles (example of a study in humans is presented on Figures 2 and 3), blood, lungs, heart, and so forth. However, other set of data adds that central and peripheral inputs can modulate the central command, and even that parallel motor activation is not obligatory for the central command control of cardiovascular responses. This was demonstrated, for example, by manipulation of effort sense during exercise by hypnosis. Morgan et al. were the first to use this method [61]. In this study, subjects exercised at a constant workload, and when hypnotized they were told that the load was light, moderate, or heavy. As a result, cardiorespiratory responses and effort sense were higher during hypnotic suggestion of a heavy load but were not different between light and moderate hypnotic suggestions. Subsequently, Williamson et al. used single-photon-emission computed tomography (SPECT) to measure regional cerebral blood flow during exercise under hypnosis [58]. Their findings corroborated those from Morgan et al., since increase in effort sense induced by hypnosis during constant-load exercise enhanced cardiovascular responses, which was associated with activation of the insular cortex, thalamus, and anterior cingulate cortex. Conversely, decreases in effort sense induced by hypnosis did not reduce cardiovascular responses below the level required to sustain metabolic needs of exercise, even though brain activation decreased. These evidences suggested that the central command can operate independent of actual motor activation, and that afferent input from the exercising skeletal muscle determines the magnitude of cardiovascular response required to sustain a given metabolic demand when effort sense is reduced below a critical level.

Corollary discharge from motor direct to sensory centers during exercise may be involved in the effort sense mediation of central command activity. This hypothesis is based, for example, on data from invasive stimulation of motor areas during brain surgery [62, 63] or noninvasive stimulation by transcranial eletromagnetic induction in humans [64]. These methods showed that stimulation of certain motor areas does not produce movement but generates the perception that movement is about to occur or supposedly occurred. Hence, it has been postulated that the motor efferent activity directed to the periphery is mirrored to the sensory cortex, which aids to organize motor planning, adjust effort sense, and so forth. Although this mechanism has already been considered important for motor control, and its network has been partially described [65], it is still largely unknown if it contributes to the central command regulation of autonomic responses to exercise [66]. Additionally, the central command may also be determined by previous experience, hedonistic inputs, mental state, and so forth, which requires further investigation [66].

The specific contribution of afferent feedback from skeletal muscles to central command modulation in humans have been investigated by Amann et al. via attenuation of afferent feedback from skeletal muscles during a closed loop exercise task (i.e., time trials were subjects that regulated the power output to achieve the best possible time) [67]. Epidural anesthesia with lidocaine aimed to attenuate all types of afferent feedback from skeletal muscles. Although it also attenuated voluntary and induced efferent muscle activation, relative integrated electromyogram of the vastus lateralis increased, pedal forces were maintained, while ventilation increased out of proportion to carbon dioxide production, and HR and blood pressure increased out of proportion to power output and oxygen consumption. These results indicated that central command activation was regulated by afferent feedback from skeletal muscles, where afferent inputs inhibited the central command. Later, to overcome the limitation of efferent motor traffic attenuation, spinal selective blockage of *μ*-opoid receptors with fentanyl was used to attenuate feedback from type III and IV fibers without affecting the force-generating capacity of skeletal muscle during a constant workload cycling bout at 80% of peak workload [68]. Using this approach, authors found muscle activation increased more than placebo, suggesting higher central motor drive. However, cardiorespiratory responses were out of proportion to power output, which probably caused a reduction in time to task failure on an open loop exercise task (i.e., a constant workload bout where subjects had to exercise as long as possible).

Thus, central command functioning without adequate feedback from peripheral muscles seems to operate unsatisfactorily. A limitation of these studies is that central motor drive was estimated from integrated electromyogram, which has many shortcomings (e.g., physiological factors: the signal depends not only on the central motor drive but also on the depression and/or facilitation of corticospinal synapses on motoneurons and muscle membrane excitability; nonphysiological factors: motion artifacts and contamination by nearby muscle's electrical activity) [69]. Although an anatomical connection between thin fiber muscle afferents and descending inputs from central command has been described [70–72], it is still unclear whether these interactive effects are functionally redundant under demanding whole body physiological exercise, as has been suggested for other types of exercise and experimental models [39]. Nevertheless, it is worth noting that independent of the origin of the central command and how it operates (feed-forward and/or feedback), its activation leads to vagal withdrawal [50, 51] and sympathoexcitation [51, 52].

Cardiovascular responses to exercise are altered in several diseases. Many studies have investigated the contribution of muscle afferences. However, few studies to date have investigated the role of central command. Koba et al. employed decerebration in rats, and then electrically stimulated the mesencephalic locomotor area, inducing fictive locomotion [73]. They noted that renal and lumbar sympathetic nerve activity increased exaggeratedly in rats with heart failure during intense stimulation of the mesencephalon. In humans, only indirect evidence about the central command is available. Negrão et al. found that patients with severe heart failure presented enhanced sympathoexcitation to mild voluntary handgrip exercise (10% of the maximal voluntary handgrip contraction) [74]. Nevertheless, in this situation it is expected that both central command and muscle mechanoreflex are activated, which limits to tease out the exact contribution of the central command. Similarly, Carrington et al. evaluated cardiovascular responses to voluntary exercise, electrically evoked contraction, and postexercise occlusion in patients with heart failure [75]. The authors attempted to deduct the responses provoked by mechanoreceptors and metaboreceptors from those observed during voluntary exercise. Although this approach shed some insights, it is an indirect approach to elucidate the role of the central command in patients with heart failure.

Summing up, the central command is traditionally considered a feed-forward mechanism, which consists of neural impulses from the motor cortex that irradiate to autonomic neurons in the brain stem, leading to parasympathetic withdrawal and sympathetic activation. In addition, recent data suggest that inputs from peripheral afferences modulate the central command activation and that mechanisms related to perceived effort are involved in the central command operation. Altered activation of the central command seems to participate on deregulated cardiovascular responses, though few studies have investigated this issue up to now.

## 3. Baroreflex

Historically, there has been some controversy whether the strength or gain of the arterial baroreflex is reduced during exercise inasmuch as in this setting both HR and arterial pressure rise. Some early studies concluded that the strength or gain of the arterial baroreflex steadily declines as exercise workload rises [76, 77]. These investigators examined the changes in ECG R wave interval (RRI) in humans in response to transient, rapid changes in arterial pressure induced by bolus injection of vasoactive drugs and found that the slope of the RRI—arterial pressure relationship (an index of baroreflex strength)—fell from rest to mild exercise and became very low as workload increased to heavy levels. However, subsequent studies using different techniques concluded that the reflex gain is sustained during exercise. For example, Melcher and Donald [78] used an isolated carotid sinus technique to examine the full range of baroreflex control over MAP and HR in dogs and concluded that the baroreflex is reset to a higher arterial pressure as workload increases. Similar conclusions were reached from human studies using devices which manipulated pressure at the carotid baroreceptors (for review see [79]).

The most likely explanations for these different conclusions are both the methods of stimulation of the baroreflex (transient versus steady-state) and the methods of analysis of the baroreflex responses (HR versus RRI, see below) [80]. Chronotropic responses to rapid transient changes in arterial pressure are predominately mediated via changes in parasympathetic tone which can elicit nearly instantaneous bradycardia/tachycardia. In contrast, the sympathetic component takes much more time to fully develop and even more so to diminish [81]. In humans (as well as canines) at rest, the heart is under strong parasympathetic control with only a small amount of tonic sympathetic activity [33, 82, 83]. As workload increases, these reverse and at high workloads little (but still some) tonic parasympathetic activity remains and sympathetic activity is greatly elevated. Therefore, the ability to elicit rapid chronotropic responses may be reduced as baseline parasympathetic activity decreases. Several studies using the spontaneous baroreflex method (observation of rapid, parasympathetic mediated chronotropic responses to spontaneous changes in arterial pressure) have shown reduced baroreflex strength during exercise [35, 84]. A further confounding issue is that the relationship between HR and RRI is highly nonlinear such that a given change in the absolute value of HR at rest translates into a much larger change in RRI than the same change in HR during exercise when baseline is markedly different (see [80] for review). In contrast to these transient stimuli, when the change in arterial pressure is sustained for sufficient time to allow full expression of both the sympathetic and parasympathetic components, similar reflex HR responses are observed at rest and during exercise (albeit if analyzed as RRI, the responses would appear reduced) [78, 83].

Neither HR nor RRI is the primary variable sensed and controlled by the arterial baroreflex. Baroreflex control over chronotropicity (as well as inotropicity) is a means to the end of controlling arterial pressure via changes in CO. However, changes in HR do not always result in changes in CO. Studies employing the spontaneous baroreflex technique showed that only about one-half of the baroreflex mediated changes in HR actually cause changes in CO at rest and during exercise this transfer is decreased even lower [85, 86]. At rest, there exists a wide range of HR over which sustained, steady-state changes in HR yield no change in CO due to reciprocal changes in SV [87, 88]. Thus, rapid parasympathetic-mediated changes in HR may alter CO transiently, but in the steady-state these changes in CO may wane due to reciprocal changes in SV likely caused by translocation of blood volume into and out of the thorax which alter ventricular filling [89]. Collectively, these observations question the utility of gauging baroreflex strength via the ability to change HR since if these changes in HR are ineffective in changing CO, then these responses do not contribute to arterial pressure regulation.

**Figure 4:**
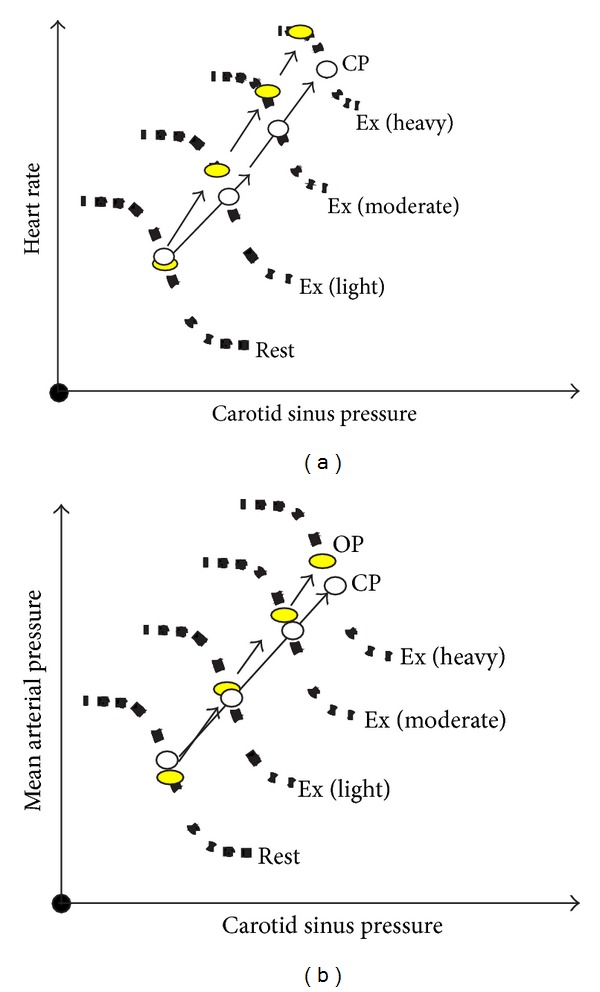
Baroreflex stimulus—response relationships at rest and mild through heavy exercise. OP: operating point, CP: centering point. Note that the reflex is reset upwards and to the right in response to exercise (adapted studies from Dr. Peter Raven's laboratory, see [79]).

In a classic study, Melcher and Donald [78] used a reversible isolated carotid sinus preparation in chronically instrumented dogs to assess the full range of baroreflex stimulus-response relationships at rest and during graded treadmill exercise. They found that the gain of the baroreflex was unaffected by exercise, instead the relationship between carotid sinus pressure and MAP or HR was shifted upwards and to the right (see Figure 4). Qualitatively similar findings were subsequently observed in humans using neck suction/pressure [90]. There are subtle but significant differences between the responses in HR versus MAP. Most often baroreflex responses are modeled as a sigmoidal relationship and therefore gain varies continuously as a function of pressure at the baroreceptors and gain highest in the center of the curve (typically at or near resting pressure) and falls toward essentially zero as pressure rises or falls outside of the response range (although a case has been made that the middle area of this relationship is essentially linear) [91]. At rest, for both HR and MAP, the operating point (OP) of the baroreflex (the prevailing level of pressure) resides very near the centering point (CP) of the reflex (the point of maximal gain, see Figure 4). However, as workload increases, the OP shifts away from the CP. This is much more dramatic for HR. As workload approaches maximal levels, the OP for HR shifts towards the upper left, flat portion of the stimulus response relationship and HR cannot increase further with a fall in pressure at the baroreceptors (Figure 4(a)). In contrast, when the baroreflex responses are analyzed in terms of MAP, this shift of the OP away from the CP is much less and the reflex is still able to buffer arterial pressure in the face of a hypotensive challenge (Figure 4(b)).

**Figure 5:**
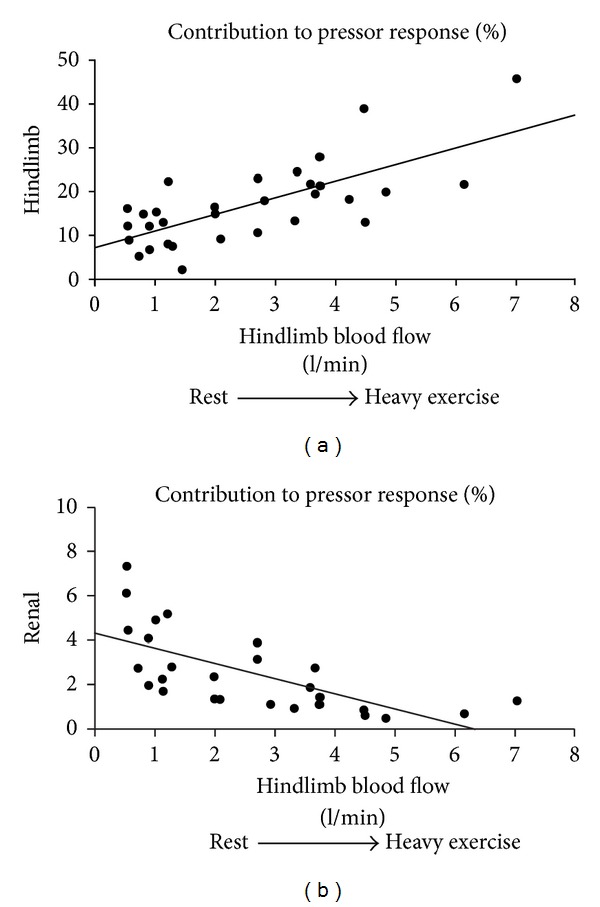
Importance of vasoconstriction in the hindlimbs versus the kidney in mediating the pressor response to carotid baroreceptor unloading at rest (low hindlimb blood flow) and during progressively increasing exercise intensities (progressively higher hindlimb blood flows). Adapted from [92].

Studies in both dogs and humans have shown that the major mechanism utilized by the arterial baroreflex to control arterial pressure at rest and during exercise is modulation of peripheral vasoconstriction [90, 92]. Although transient changes in CO occur in response to initial carotid sinus perturbations, these changes are not sustained and in the steady state most of the reflex change in pressure is due to the vascular responses [90]. As workload increases, blood flow to the active skeletal muscle becomes a larger fraction of CO and total vascular conductance. At higher workloads, effective regulation of arterial pressure through vascular responses can only occur via control of vascular conductance in the active skeletal muscle [93].  Figure 5 shows the fraction of the pressor response to carotid baroreceptor unloading due to vasoconstriction in the hindlimbs versus the kidneys at rest and during graded treadmill exercise. As workload rises and the baseline level of hindlimb blood flow increases, the contribution of vasoconstriction in the hindlimbs to the pressor response steadily rises. Although at rest and at each workload similar changes in renal vascular conductance occurred with carotid baroreceptor unloading, because renal blood flow becomes a progressively smaller fraction of CO as workload rises, the contribution of renal vasoconstriction to pressure regulation becomes negligible at high exercise intensity. Extrapolating the hindlimb responses to the rest of the active skeletal muscle in the body, over 80% of the rise in arterial pressure during baroreceptor unloading at high workloads is due to vasoconstriction in skeletal muscle [92].

Resetting of the arterial baroreflex may occur from stimulation of skeletal muscle afferents and/or activation of central command [94, 95]. As workload rises, both mechanisms would provide a progressive input to central brainstem neural networks controlling baroreflex resetting via either ascending feedback input from skeletal muscle mechanosensitive and metabosensitive group III and IV afferents or via descending feed-forward input from central command with increasing effort. Resetting of the baroreflex may be more than just permissive to the rise in MAP and HR with exercise as after baroreceptor denervation MAP falls at the onset of exercise and is labile [96]. This is likely due to unrestrained vasodilation in the active skeletal muscle [97]. However, as workload rises and powerful reflexes arising from activation of skeletal muscle afferents engage, the arterial baroreflex may act to prevent excessive sympathoactivation [97–99]. Left unbuffered by the arterial baroreflex, this sympathoactivation during exercise could cause vasoconstriction in the active skeletal muscle and even within the coronary circulation [100–103]. Such a scenario could limit both peripheral perfusion and ventricular performance which could ultimately lead to exercise intolerance.

## 4. Carotid Chemoreflex

The hydrogen ion concentration in the brain extracellular fluid and the arterial blood gas composition are monitored, respectively, by receptors within the central nervous system (central chemoreceptors) and in conduit arteries (peripheral chemoreceptors) [104]. The central chemoreceptors include neurons located at the ventrolateral medulla and other distributed neurons (e.g., nucleus of the solitary tract, retrotrapezoid nucleus, raphe, pre-Bötzinger complex, locus coeruleus, and hypothalamus) [105]. They are activated by hypercapnia and inhibited by hypocapnia, which is mediated by variation in hydrogen ion concentration caused by CO_2_ diffusion from blood to the cerebral spinal fluid [106]. The peripheral chemoreceptors are highly perfused structures located at the carotid sinus (carotid body) and the aortic arch [101]. Both are activated by hypoxia and inhibited by hyperoxia [106]. Aortic chemoreceptors responsiveness to arterial partial pressure of O_2_ (PaO_2_) is markedly smaller than the carotid chemoreceptors [107, 108]. In addition, carotid body denervation almost abolishes the ventilatory [109, 110] and sympathetic [111] response to hypoxia in animals. More importantly, bilateral carotid body tumor resection in humans abolishes the ventilatory drive to normocapnic hypoxia. Thus, the carotid chemoreceptors have been considered the major O_2_ sensor in the body [112].

Hypoxia and hypercapnia may occur in patients with cardiopulmonary disease during exercise [104] or may be present even at rest and get worst during exercise in these patients [106]. Hypoxia also occurs at rest and during exercise in healthy subjects during exposure to high altitude [113]. Consequently, in these situations, there is consensus that central and peripheral chemoreceptors play a key role in the physiological adjustment during exercise [104] including autonomic responses (i.e., vagal withdrawal and sympathoexcitation). On the other hand, the partial pressure of O_2_ in the arterial blood is well maintained in healthy subjects during exercise in normoxia that would not suggest a direct stimulus for the carotid chemoreceptors to contribute for the autonomic response to exercise. Moreover, the partial pressure of CO_2_ is quite stable during moderate intensity exercise, and it decreases during heavy and severe exercise due to hyperventilation, which is not a direct stimulus for the central ventilatory drive [104]. Consequently, although the chemoreflex contribution for the autonomic response to exercise has been traditionally emphasized for patients with hypoxia/hypercapnia and exposure to high altitude, its role in the autonomic responses during normoxic exercise has been overlooked [114]. However, by means of studies conducted in dogs and humans that started at the University of Wisconsin (mentored by Dr. Jerome Dempsey) [115, 116] and continued at the University of Alberta [117], Stickland et al. have shown that the carotid chemoreceptors contributes for roughly one-third of the sympathetic vasoconstrictor outflow during exercise in normoxia. These evidences highlighted that the carotid chemoreflex is one of the mechanisms that participates in the neural control of circulation during exercise, which is detailed hereafter.

The contribution of the carotid chemoreceptors for the sympathetic restraint of skeletal muscle blood flow was firstly investigated in chronically instrumented dogs [116]. Close intra-arterial infusions of hyperoxic saline or dopamine were conducted at rest and during mild treadmill exercise, with the catheter tip positioned at the bifurcation of the common carotid artery. These infusions aimed to inhibit the carotid chemoreceptors and were done transiently, instead of continuously, to avoid compensatory mechanisms and systemic effects of infusions [118, 119]. Resting blood flow and conductance did not change in healthy dogs, but during mild exercise, inhibition of carotid chemoreceptors caused an abrupt vasodilation in the hindlimb. This result indicated that carotid chemoreceptors restrained the blood flow during exercise under normoxia. Blockade of *α*-adrenergic receptors via infusion of phentolamine in the abdominal aorta abolished the vasodilatory response, which suggested that restriction of muscle blood flow during exercise was mediated by the vasoconstrictor sympathetic outflow. Subsequently, carotid body was denervated in some dogs to confirm the participation of the carotid chemoreceptors. After denervation vasodilation by infusion of dopamine or hyperoxic saline was abolished. Chronic heart failure was then induced in some of these healthy dogs by chronic rapid cardiac pacing, and the closed carotid infusions were repeated. Inhibition of carotid chemoreceptors with dopamine or hyperoxic saline caused vasodilation both at rest and during exercise in dogs with CHF, and this response was abolished by *α*-adrenergic blockade. Thus, the carotid chemoreceptors restrained the blood flow at rest and during exercise in dogs with CHF under normoxia via the sympathetic vasoconstrictor outflow.

**Figure 6:**
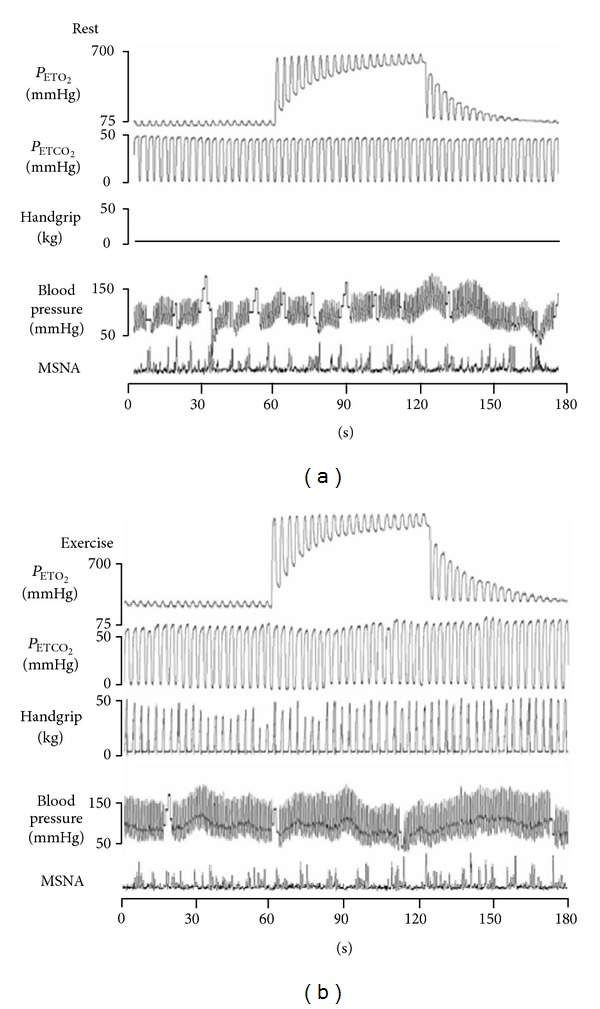
Representative trace of a subject receiving hyperoxia at rest (top) and during exercise (bottom). Hyperoxia was administered from 60 s to 120 s. There was no significant change on muscle sympathetic nerve activity (MSNA) during hyperoxia administration at rest. On the other hand, hyperoxia administration during exercise reduced MSNA by roughly 35%. This showed that the carotid chemoreflex was sensitized, and that it contributed to the MSNA increase during exercise. *P*_ETO__2_, end tidal oxygen pressure; *P*_ETCO__2_, end tidal carbon dioxide pressure.

Afterward these findings were translated to healthy humans [116, 117]. In one study [116], carotid chemoreceptors were inhibited by inhalation of hyperoxic gas (see Figure 6). The inhalation was repeatedly administered for 1 min in the middle of 3 min of recording at rest and exercise, while breathing rate, tidal volume, and end tidal partial pressure of CO_2_ were carefully controlled at standardized levels. Exercise consisted of bilateral rhythmic handgrip (1 s contraction and 2 s relaxation) at 50% of maximal voluntary contraction. Hyperoxia did not change muscle sympathetic activity (MSNA) at rest. Conversely, MSNA decreased 35% when hyperoxia was administered during exercise. These results corroborated those from healthy dogs, confirming by direct nerve activity recording that the carotid chemoreceptors reflex is functional in humans during exercise in normoxia. In another study [117], carotid chemoreceptors were inhibited by inhalation of hyperoxic gas and/or intravenous infusion of dopamine, at rest and during constant load double leg kicking exercise, to investigate if carotid chemoreceptors regulate blood flow during exercise in healthy humans. Hyperoxia did not change resting femoral artery blood flow and conductance but increased femoral artery blood flow and conductance during exercise in the first 45 s of continuous hyperoxia administration for 2 min. After 45 s of hyperoxia, femoral blood flow and conductance decreased toward the values observed during normoxic exercise. When hyperoxia was administered during exercise on top of a background dopamine infusion (i.e., carotid chemoreceptors were previously inhibited by dopamine), the hyperoxia impact on femoral blood flow and conductance was abolished. Hence, these findings confirmed those from healthy dogs [115] and added that carotid chemoreceptors contribute to skeletal muscle blood flow regulation during normoxic exercise in healthy humans.

Earlier studies from other groups did not observe the important contribution of the carotid chemoreceptors described by Stickland et al. [115–117]. The reason for that seems to be attributed to crucial methodological details that differed among studies. For example, a common method to inhibit the carotid chemoreceptors, particularly in humans, is the administration of hyperoxia. Previously hyperoxia was administered continuously during a period at rest (from 3 min to 15 min) until a steady cardiorespiratory response was attained [120, 121]. Then resting and exercise data were collected. This might have had a large influence on results, since some minutes of exposure to hyperoxia have a secondary effect on chemoreflex function [122–124] presumably due to production of reactive oxygen species [119, 125]. This was observed in the context of carotid chemoreceptors contribution during exercise in normoxia, when hyperoxia administration started during exercise and resumed during 2 min [116]. In this case, the carotid chemoreceptors contribution was observed only during the first 45 s of hyperoxia administration but was absent after that. Therefore, studies aiming to deepen the understanding of the carotid chemoreceptors to the neural control of circulation during exercise in normoxia should use transient inhibition of carotid chemoreceptors [119] as well as control for confounding factors such as breath pattern [126–128], partial pressure of CO_2_ [107, 108, 129], and sensitization of the carotid chemoreceptors by previous hypoxia exposure [130].

Many questions emerged after the demonstration that the carotid chemoreceptors contribute to the restraining of blood flow during exercise in normocapnic normoxia via the sympathetic vasoconstrictor outflow. An important question regards what mechanisms sensitize the carotid chemoreceptors during exercise in healthy animals and humans under normoxia. There is evidence that the carotid chemoreceptors have a polymodal sensory function [106], being stimulated not only by PaO_2_ but also by potassium [131], angiotensin [132], cathecolamines [107, 108], adenosine [133], oxidative stress [134], and temperature [135]. Since all of these factors change during exercise, it is possible that they increase the sensitivity of the carotid chemoreceptors, even with no chance in the PaO_2_. It is also likely that neural mechanisms (i.e., central command, mechanoreflex and/or metaboreflex) interact with afferences from the carotid chemoreceptors, which changes the central processing of signals from carotid chemoreceptors without change on the arterial partial pressure of O_2_. Another thought-provoking question regards the contribution of the carotid chemoreceptors for autonomic responses to exercise in some diseases. For example, obstructive sleep apnea [136], hypertension [137], and chronic heart failure [134] are accompanied by enhanced peripheral chemoreflex sensitivity. Moreover, there is recent and strong data that the carotid chemoreceptors are involved in the development of hypertension in spontaneous hypertensive rats [138], since carotid body denervation was mechanistically accompanied by resetting of renal sympathetic nerve activity-baroreflex function curve, sensitization of the cardiac baroreflex, and reduced T-lymphocyte infiltration into the aorta and brainstem [139]. In rats with chronic heart failure, carotid chemoreceptors selective ablation improved survival, which was associated with reduced presympathetic neuronal activation and normalized indices of resting sympathetic outflow and baroreflex sensitivity [140].

In summary, recent experimental approaches have changed the understating of the carotid chemoreflex contribution to the autonomic responses during exercise in normoxia and normocapnia. Recent data suggest that carotid chemoreceptors are sensitized during exercise and so mediate about one-third of the increase in sympathetic activity during exercise in healthy humans. Therefore, it is likely that the carotid chemoreceptors have an important and still undetermined contribution for the vagal withdrawal and sympathoexcitation during exercise also in patients with cardiovascular diseases, which might be physiologically and clinically important, deserving further investigation.

## 5. Exercise Pressor Reflex

Alam and Smirk [141, 142] demonstrated in their seminal works that dynamic calf exercise evoked an increase in blood pressure, which was maintained by circulatory arrest after cessation of exercise. Then, once blood flow was restored, blood pressure fell. These experiments demonstrated that metabolic reflex originating in skeletal muscle can mediate cardiovascular adjustments to exercise, the so called “metaboreflex.” Later, it has been demonstrated that mechanical changes in muscles and tendons can also elicit cardiovascular responses, a reflex termed “mechanoreflex” [143, 144]. These two reflexes of muscular origin concur in generating the exercise pressor reflex.

Afferent fibers from skeletal muscle are classically subdivided into four groups (I to IV) on the basis of their anatomical and electrophysiological characteristics [145]. Slow conducing nerve fibers of groups III and IV are thought to be excitable by mechanical and chemical stimulation [146, 147].

The majority of group III afferents are rapidly excited by mechanical distortion of their receptive field. Thus, type III nerve afferents probably act as mechanoreceptors and contribute to the initiation of the exercise pressor reflex [148]. On the contrary, the firing behavior found in several fibers of group IV appear especially well suited to function as metabolic receptors [149, 150]. In fact, their onset latency is a period of time compatible to allow the metabolic products of contraction to accumulate in a muscle undergoing contraction. Moreover, the firing rate of these group IV fibers gradually increased as the contraction lasted, due to buildup of metabolites in the contracting muscles. However, it has been reported that a subpopulation of group III/IV afferent fibers are polymodal; that is, they respond to both mechanical and chemical stimuli [143, 148]. Moreover, evidence suggests that mechanoreceptors can be sensitized by metabolites [149].

From the above results it seems clear that receptors within muscles gather information concerning the mechanical (muscle length and strain, tissue compression,deformation due to contractions) and metabolic (amount of metabolites accumulation) conditions of the muscles involved in the exercise being performed. This information is then provided to the cardiovascular controlling areas, which operate the hemodynamic adjustments in order to regulate blood flow on the basis of the status of contracting muscle.

Groups III and IV muscle afferents project to the dorsal horn of the spinal cord. Less is known about the central pathways of the exercise pressor reflex, even though it seems that the* medulla oblongata* is essential for its expression [31, 141, 149, 151–153]. It appears that neuronal cells responsive to the exercise pressor reflex are located in the* nucleus tractus solitarius*, rostral ventral medulla, caudal ventrolateral medulla, lateral tegmental field,* nucleus ambiguus*, and the ventromedial region of the periaqueductal grey [154].

Concerning the cardiovascular response generated by those nerve fibers which are activated by metabolites accumulation (i.e., the “metaboreceptors”), they are thought to be sensitive to several substances such as lactic acid, potassium, bradykinin, arachidonic acid products, ATP, diprotonated phosphate, and adenosine [154–156]. It is remarkable that several of these metabolites are also involved in the phenomenon known as remote preconditioning, which refers to the possibility of preconditioning the heart, that is, to render the heart more resistant to sustained ischemia, by causing ischemia in a remote organ. It has been proposed that remote preconditioning is initiated by humoral factors released into the blood and transferred to the heart where they trigger cardioprotection. Even though the exact mechanisms involved are still unknown, key molecules appear to be adenosine and bradykinin [157]. Moreover, it has been recently demonstrated that dialysated plasma from humans undergoing exercise reduced infarct size in isolated rabbit hearts after ischemia-reperfusion injury [158, 159]. Authors of these investigations concluded that exercise can induce cardioprotection by triggering remote preconditioning and that this cardioprotection was at least partially mediated by systemic release of one or more humoral factors. It is therefore conceivable to hypothesize that the release of metabolites eliciting the metaboreflex may also trigger preconditioning. To the best of our knowledge this hypothesis has never been tested.

As concerns receptors, recent evidence suggests that cannabinoids, *μ*-opioid, acid-sensing ion channels, transient receptor potential vanilloid 1, and purinergic ligand-gated ion channels are all candidates as potential mediators of the metabolic-induced cardiovascular response [148, 160]. It is unclear whether a threshold for metaboreceptors stimulation by muscle end-products exists or not, that is, whether metaboreflex operates only at moderate-high exercise intensities, when metabolites accumulate within working muscle, or even at mild muscle strain, when there is probably no mismatch between muscle blood supply and demand and metabolites only minimally accumulate. It has been reported that the muscle metaboreflex in humans has a threshold of around a pH of 6.9 units and that MAP increases linearly with decreasing muscle pH [160]. Besides, studies employing ^31^P nuclear magnetic resonance spectroscopy found that decrements in intramuscular pH were coupled to the rise of sympathetic nerve activity, thus suggesting that an event associated with glycolysis and lactate production may be important in activating the reflex [161–163]. These findings are consistent with the concept that the metaboreflex is activated whenever blood flow to contracting muscles is insufficient to warrant both oxygen delivery and metabolite washout [31, 34, 163]. According to this viewpoint, the metaboreflex acts to correct any mismatch between muscle blood flow and metabolism by superimposing to the central command activity.

However, it has also been demonstrated that group IV fibers of cat muscle are responsive to a low level of exercise, that is, when there is no mismatch between blood delivery and metabolic needs of working muscle [149]. The response at low level stimulation was interpreted with the fact that the metaboreflex plays a role in cardiovascular regulation even when there is sufficient O_2_ delivery to the muscle and a mismatch between muscle flow and metabolism is not yet detectable. This is in accordance with the findings of Strange and coworkers [30] who demonstrated the essential role of the metaboreflex in humans in order to reach normal blood pressure response even for mild exercise eliciting a HR below 100 bpm. Therefore, it is possible that the metaboreflex is evoked even at low workloads, without any apparent accumulation of end-products of metabolism. According to this viewpoint, metaboreflex is responsible for a tonically active feedback to the cardiovascular control areas that starts working whenever the metabolism is activated by muscle contraction [68, 164].

### 5.1. Hemodynamic Effects of Metaboreflex and Mechanoreflex Activation 

The typical hemodynamic response to metaboreflex activation is an increase in arterial blood pressure [31, 34, 36, 165]. This cardiovascular adjustment is thought to be primarily achieved by increasing SVR because of peripheral sympathetic vasoconstriction [31, 34]. The reflex effect on HR is supposed to be small or absent, and this fact led some authors to speculate that metaboreflex has little or no effect on CO. However, it should be considered that the effect on HR strongly depends on the setting of metaboreflex activation. In fact, two approaches have been used to study the relationship between hemodynamics and metaboreflex: (1) reducing muscle blood flow during effort, or (2) reducing blood flow at the cessation of effort by postexercise ischemia in order to trap metabolites produced during previous muscle contraction [33, 166, 167]. This latter protocol is used in order to isolate the effects of metaboreflex activation from those due to central command and mechanoreflex.

While the activation of muscle metaboreflex during exercise can elicit HR response through an increase in sympathetic activity towards sinus node, during postexercise ischemia the rise of sympathetic activity is masked by the concomitantly enhanced parasympathetic outflow due to the loss of central command [167]. Parasympathetic tone during postexercise ischemia can be further increased by arterial baroreflex, which responds to the metaboreflex-induced increase in blood pressure by buffering the elevated sympathetic drive to the heart and arteriolar vessels [35]. Thus, if metaboreflex is activated by postexercise ischemia, the elevated sympathetic activity to sinus node is counteracted by enhanced parasympathetic tone due to the withdrawal of central command and to the sympathetic-buffering effect of baroreflex activation. The resulting effect is that HR decreases despite the fact that sympathetic tone to the heart is maintained high [33, 168].

While the effect of metaboreflex upon SVR and HR is well established and accepted, less is known about its influence on central hemodynamics, that is, CO, SV, myocardial contractility, and cardiac preload. Much evidence suggests that the activation of metaboreflex can actually also affect central hemodynamics. In particular, it was found that muscle metaboreflex in dogs was able to increase ventricular performance which, in turn, maintained SV constant despite the concomitant increase in HR and the consequent reduction in diastolic time and cardiac filling [169]. Similarly, it has been reported that myocardial contractility and SV in humans can be increased during metaboreflex activation caused by postexercise ischemia [36, 170–175]. This hemodynamic response aims at maintaining CO despite the rise in after-load that takes place in this condition as a consequence of vasoconstriction [68]. However, it should be considered that conflicting results exist in scientific literature on the role of SV and CO in mediating blood pressure response to metaboreflex and that some papers have reported that SV does not significantly participate in this response [168, 176, 177].

Various reasons might account for this different outcome: firstly, it should be noted that a rhythmic forearm exercise was employed in studies reporting SV response to metaboreflex, while in the experiments reporting a pivotal role of peripheral vasoconstriction, a static exercise (handgrip) was employed, which is expected to induce a substantially greater pressor response and an increase in after-load than dynamic handgrip. This fact could have limited the SV increment, thus explaining the different outcome. Secondly, the protocols employed were very different in terms of workloads and duration. The importance of increasing cardiac contractility and SV appears particularly when the metaboreflex is evoked during postexercise ischemia, that is, when bradycardia occurs, since in this circumstance the cardiovascular apparatus can rely only on the enhanced myocardial contractility to maintain SV and CO constant in response to vasoconstriction [36].

It has also been proposed that muscle metaboreflex is capable of increasing cardiac filling pressure through splanchnic vasoconstriction and venoconstriction which expel blood volume into the central circulation [176, 178, 179]. This fact produces a sort of blood volume “centralization” in order to support SV and CO.

**Figure 7:**
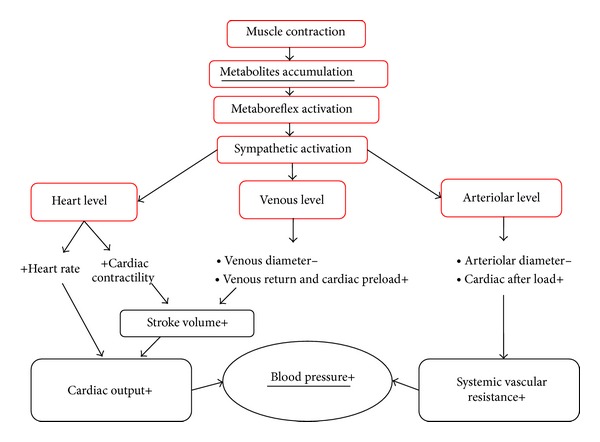
Mechanisms through which metaboreflex operates to increase blood pressure: heart rate, contractility, afterload, and preload modulation. See text for more details.

Thus, it appears, at least in healthy individuals, that hemodynamic response to metaboreflex activation is a highly integrated phenomenon and that complex interplay between HR, cardiac performance, preload, and after-load occurs when this reflex is recruited [36, 165, 172, 175, 176, 178, 180] and that cardiovascular adjustment is not solely the consequence of an increase in SVR.  Figure 7 shows the putative mechanisms through which metaboreflex acts to increase blood pressure.

It is noteworthy that the strategy chosen to achieve the blood pressure response appears to depend on the presence of cardiac reserve, which represents the possibility of the heart to increase contractility and in turn SV. If cardiac reserve is still available (i.e., if the contractility was not fully used during exercise), then the metaboreflex-induced pressure response relies mainly on CO; differently, if cardiac reserve is no longer available to increase SV and CO, as it is during and/or after strenuous efforts, then the response relies on peripheral vasoconstriction and SVR increase. These findings suggest that the intensity of exercise possibly dictates the mechanism by which the blood pressure response is elicited by metaboreflex [36, 181].

A debating point is whether or not the metaboreflex acts as a flow-raising mechanism to preserve muscle perfusion during exercise. In humans, during moderate-heavy dynamic exercise with large muscle mass the metaboreflex may function to curtail excessive increases in muscle blood flow due to metabolites accumulation and to prevent excessive vasodilation in the contacting muscle. This fact maintains systemic perfusion pressure. Thus, in this setting the metaboreflex probably acts in opposition to vasodilation and to preserve systemic pressure rather than to enhance blood flow to active muscle. In humans, there is only limited evidence for a flow-raising effect of the metaboreflex during moderate dynamic exercise. Probably, the flow-raising effect occurs during submaximal exercise, when there is a sufficient reserve of CO, and when there is a reduced production of vasodilating metabolites in the exercising muscle [182].

A particular aspect of metaboreflex activation is related to its effect on the coronary circulation. Since the metaboreflex increases sympathetic discharge it may also activate coronary *α*-adrenergic receptors, which may limit metabolically induced vasodilation at the coronary tree level. In experiments conducted in dogs it has been found that activation of the metaboreflex during dynamic exercise can induce significant coronary vasoconstriction [183, 184]. Normally, metaboreflex activation elicits no coronary vasodilation despite substantial increases in cardiac work which by itself would be expected to cause coronary metabolic vasodilation (greater CO pumped against a much higher afterload). A similar finding was seen in humans during postexercise circulatory occlusion [185]. When cardiac work was lessened, significant coronary vasoconstriction occurred with metaboreflex activation [183] which was reversed to coronary vasodilation after *α* adrenergic blockade [184]. These findings underscore that the metaboreflex may exert a robust vasoconstriction which may restrain coronary vasodilation. In subsequent investigations it has also been found that sympathetic restraint of coronary vasodilation functionally limits increases in ventricular contractility [100]. Moreover, in dogs with heart failure it has been observed that during the metaboreflex the inability to raise ventricular contractility was not only due to ventricular dysfunction but was also to be ascribed to coronary vasoconstriction, which limited myocardial perfusion [101]. However, to the best of our knowledge, there is no study which has investigated the effect of metaboreflex activation on coronary circulation during heart failure in the human setting. Thus, it is not known whether these results in canine model of metaboreflex activation can be applied also in humans.

Likewise, as reported for the metaboreflex, the mechanoreflex has been demonstrated to be able to elicit cardiovascular reflex. Mechanical distortion of receptive fields of sensory nerve endings in contracting muscle may evoke the exercise pressor reflex in humans [186, 187]. In detail, it appears that the main effect of mechanoreflex activation is an inhibition of cardiac vagal tone which, similarly to what has been reported for central command, produces a rapid and sustained increase in HR at the onset of exercise [143, 184]. However, in humans the mechanoreflex is more difficult to isolate than metaboreflex as muscle contraction is accompanied by both central command and metaboreflex activation. Moreover, evidence suggests that mechanoreceptors are sensitized by metabolites accumulation, thereby enhancing sympathetic response arising from their activation and making it difficult to isolate pure mechanostimulation from metabostimulation [154].

The importance of exercise pressor reflex dysregulation in the genesis of hemodynamic abnormalities observed during exercise in some cardiovascular and metabolic diseases such as heart failure, hypertension, heart transplant, obesity, metabolic syndrome, and types 1 and 2 diabetes mellitus has been underlined in several recent studies [174, 188–193]. Moreover, abnormal cardiovascular response has also been seen in spinal cord injured patients [179]. From these investigations in the clinical setting it can be argued that impairment in one or more of the cardiovascular parameters modulated during the metaboreflex (i.e., chronotropism, inotropism, cardiac preload, and after-load) leads to altered hemodynamic response [188, 189, 194–196]. Further studies are warranted to better clarify the importance of exercise pressor reflex in the cardiovascular regulation of these pathologies.

In summary, from available data it seems that in healthy subjects the exercise pressor reflex operates by adjusting all the four hemodynamic modulators to achieve the target blood pressure response: HR, contractility, preload, and after-load. An impairment in one or more of these modulators leads to abnormal hemodynamic adjustments to exercise.

## 6. Conclusions and Future Directions

In summary, the autonomic regulation during dynamic exercise is achieved on the basis of several inputs arising from motor cortex, arterial, and skeletal muscle receptors. The cardiovascular controlling centers integrate this information and reflexively generate the hemodynamic adjustments. In healthy individuals, sympathetic activity to cardiovascular apparatus augments and prevails over parasympathetic tone, thereby increasing HR and myocardial contractility and constricting the vascular beds of organs and tissues not involved in exercise. Some dysregulation in these mechanisms has been demonstrated in various diseases, mainly circulatory and metabolic diseases. However, it is likely that many other pathologies not directly linked to the cardiovascular apparatus, such as neural, muscular, inflammatory, and sedentary lifestyle, can affect the cardiovascular response during exercise operated by these reflexes. Thus, further investigation in this field is warranted. Moreover, it is important that future studies take into account the possible effects of pharmacological therapy on the correction of the dysregulation of these reflexes.
